# Age‐Related Changes in Marmoset (*Callithrix jacchu*s) Feeding Behavior and Physiology: Insights of Masticatory and Swallowing Functions

**DOI:** 10.1002/ajp.70070

**Published:** 2025-08-26

**Authors:** Max Sarmet, Sachiko Takehara, Priscila Sales de Campos, Kensuke Kagiyama, Yasuhiro Kumei, Christopher J. Mayerl, Laura Davison Mangilli, Jorge Luís Lopes Zeredo

**Affiliations:** ^1^ Graduate Program in Health Science and Technology University of Brasilia Brasilia Distrito Federal Brazil; ^2^ Department of Biological Sciences Northern Arizona University Flagstaff Arizona USA; ^3^ Department of Oral Health Science, Division of Preventive Dentistry, Graduate School of Medical and Dental Sciences Niigata University Niigata Japan; ^4^ Breathing Research and Therapeutics Center University of Florida Gainesville Florida USA; ^5^ CLEA Japan Inc. Gifu Japan; ^6^ Department of Pathological Biochemistry, Graduate School of Medical and Dental Sciences Tokyo Medical and Dental University Tokyo Japan

**Keywords:** animal model, Callithrix, cineradiography, healthy aging, mastication, swallowing

## Abstract

The common marmoset (*Callithrix jacchus*) is a valuable model for studying aging due to its physiological and social similarities to humans, including shared susceptibilities to age‐related diseases. However, the effects of healthy aging on marmoset mastication and swallowing are poorly understood, despite their importance for modeling human aging and understanding marmoset ecology and longevity (efficient food processing impacts foraging success and predation risk). Given their specialized diet, dental adaptations, and relatively long lifespan compared with other biomedical models commonly used, like rodents, understanding how elderly marmosets maintain feeding efficiency is particularly important, yet lifespan research on their feeding physiology is scarce. Using cineradiography (with a microfocal X‐ray source and beryllium image intensifier), we examined masticatory and swallowing physiology across the marmoset lifespan (1 month to 19 years) in 26 healthy individuals, analyzing 45 recordings (80 feeding events, 784 swallows). Our study revealed a developmental trajectory in marmoset chewing and swallowing, from infancy to old age, characterized by progressively refined handling of larger food portions and boluses. We identified distinct anatomical, functional, and behavioral differences in feeding physiology among age groups. Elderly marmosets exhibited significantly faster feeding rates than infants and adults, consuming larger portions and forming larger boluses, requiring fewer mastications and swallows, likely reflecting age‐related adaptations. Notably, old and very old marmosets showed comparable feeding efficiency, suggesting compensatory mechanisms to maintain function despite age‐related challenges (e.g., tooth loss or muscle weakness) and may contribute to longevity. The consistent pattern of esophageal retention across age groups indicates this pattern is likely typical for the species. This study establishes baseline feeding characteristics for marmosets, reinforcing their value as a translational aging model and enhancing their utility for investigating age‐related changes in human chewing and swallowing, including dysphagia. Future research should explore the underlying mechanisms and functional implications of these changes.

## Summary

- This study offers a first‐time investigation of age‐related changes in marmoset food processing (chewing and swallowing). Infants demonstrate progressive refinement of these skills, handling larger portions and boluses as they mature.
- Elderly marmosets eat larger portions with fewer chews per swallow than younger ones, suggesting they develop compensatory mechanisms to maintain swallowing efficiency despite age‐related dental limitations.
- This study expands knowledge of marmoset anatomy, feeding behaviors, and healthy lifespan regulation. The observed swallowing adaptations likely represent a natural physiological response to aging in nonhuman primates.

## Graphical abstract

This study investigated the feeding and swallowing physiology of 26 marmosets (1 month to 19 years old). Using a non‐invasive cineradiography setup, the study examined key age‐related differences in oral and pharyngeal swallowing phases across four age groups: infant, adult, old, and very old. Our findings indicate that elderly marmosets exhibit significantly different feeding behaviors, including larger food portions, fewer mastions, and larger boluses. These results provide novel insights into the adaptations and compensatory mechanisms of swallowing with age.

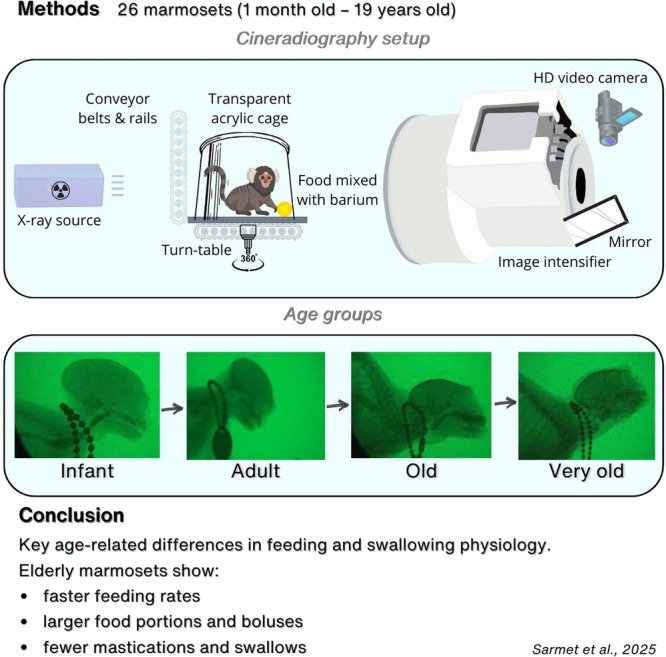

## 1 Introduction

The common marmoset (*Callithrix jacchus*) is a valuable model for aging research, exhibiting similarities to human aging in biological and social characteristics, including susceptibility to age‐related pathologies such as cancer, diabetes, arthritis, cardiovascular disease, and neurological decline (Tardif 2019; Colman 2018; Zeredo et al. 2019; Mattison and Vaughan 2017; Mansfield 2003; Takehara et al. 2019; Arruda et al. 2019). However, our understanding of healthy aging in marmosets, particularly regarding age‐related changes in mastication and swallowing, remains incomplete. Investigating these physiological changes in a controlled environment can enhance our understanding of aging in this species and potentially establish marmosets as a model for studying age‐related swallowing disorders, an especially critical task given the prevalence of swallow dysfunction in elderly populations (Ambiado‐Lillo 2024; Labeit et al. 2022).

Understanding how age impacts food processing is crucial not only for modeling human aging but also for understanding marmoset ecology, as efficient food processing influences both foraging success and predation risk (McGraw and Daegling 2012; Barros et al. 2008). The maintenance of feeding efficiency is essential for longevity, yet how elderly marmosets adapt to challenges like tooth loss and muscle weakness remains unclear. This is particularly relevant given their specialized gummivore‐insectivore diet and relatively long lifespan compared with other biomedical models commonly used in aging research (e.g., mice). In captivity, common marmosets typically live 13–16 years on average after reaching sexual maturity around 18 months (Solomon and Rosa 2014; Fleagle et al. 2024; Schultz‐Darken et al. 2016), with exceptional cases living over 22 years (Sarmet et al. 2024). Their specialized diet has led to dental and digestive adaptations for tree gouging (Coimbra‐Filho and Mittermeier 1976; Vinyard et al. 2003), including powerful jaw musculature (Mansfield 2003; Vinyard et al. 2003; Eng et al. 2009). Despite these adaptations and the marmosets' value as an aging model, research on their feeding physiology is limited.

Primate swallowing research is scarce and focused on catarrhines, with single‐subject studies in *Macaca fascicularis* (German et al. 1992) and *Macaca mulatta* (Best et al. 2015), and further *M. mulatta* studies on orofacial neurophysiology (Arce et al. 2013; Arce‐McShane et al. 2014, 2016). Callitrichine swallowing remains unexplored. While primate mastication studies have primarily focused on catarrhines, our group recently published the first detailed study of callitrichine mastication, using cineradiography to examine craniofacial development and chewing in infant marmosets (de Oliveira et al. 2022).

To address these gaps, this study examines masticatory and swallowing physiology across the marmoset lifespan, including exceptionally long‐lived individuals (14–19 years old), to provide a more complete understanding of age‐related changes. Using cineradiography, this study aims to: (1) establish baseline characteristics of masticatory and swallowing physiology in healthy captive marmosets; and (2) elucidate how these functions change with age.

## 2 Methods

We conducted this cross‐sectional study at CLEA Japan Inc., after the Animal Welfare Committee of CLEA Japan Co. Ltd. approved protocol 1652‐012CJ before study initiation. All animal care and procedures adhered to the American Society of Primatologists' Principles for the Ethical Treatment of Non‐Human Primates and complied with the Act on Welfare and Management of Animals (Act No. 105 of 1973), the primary Japanese legislation governing animal research. We did not euthanize any animals during this study.

### 2.1 Animals and Accommodation Conditions

We included in this study 26 healthy research‐naïve common marmosets (*Callithrix jacchus*, 12 males and 14 females) ranging in age from 1 month to 19 years. The youngest animal was 31 days old. Due to radiation risks, we excluded animals deemed unhealthy by the veterinarian, as well as pregnant females, from the study. We subsequently returned them unharmed to their home cages. We categorized animals into age groups based on neurobehavioral developmental stages: infants (0–20 weeks), juveniles (20–40 weeks), subadults (40–60 weeks), adults ( > 60 weeks), and olds (> 288 weeks), as defined by Schultz‐Darken et al. (2016). Recognizing a second inflection point in senescence for animals older than 14 years, as identified by Nishijima et al. (2012), we further categorized the oldest animals as “very old” in this study. Due to limited animal availability, juvenile and subadult animals could not be recorded at the time of this study. All animals originated from the CLEA Japan colony (Gifu, Japan). Colony staff housed the animals there, and the researchers performed all procedures at the colony (see Nishijima et al. 2012, for colony details). Staff housed the marmosets in stainless steel cages (75 × 39 × 55 cm; height × width × depth) with solid plating on the left and back sides, maintained at 28 ± 1°C under a 12‐h light/dark cycle, and provided ad libitum water and species‐specific chow (CMS‐1M; Feed One Co.). Staff housed infants in continuous full contact with their breeding pairs; adults were pair‐ or group‐housed in continuous full contact; and elderly marmosets were pair‐housed in continuous full contact if healthy and dentate, otherwise single‐housed for safety, preventing aggression and ensuring appropriate access to softened food (for partially dentated individuals). We withheld food on experimental days until after data collection (around 7:00 a.m.). To minimize animal stress and ensure their health and safety, we provided novel enrichment, cage furnishings, and positive human interaction, and facilitated communication between animals in different cages via visual displays, olfactory cues, and vocalizations, while concurrently performing all transient, minimally invasive procedures on healthy animals (see Material S1).

### 2.2 Oral Examination

A dental surgeon (Y. K.) performed a standard oral examination on all marmosets, recording the number of teeth and the presence of spontaneous (observed) or induced (cotton swab) gingival bleeding. An assistant manually restrained the marmosets with a wooden chopstick used to keep their mouths open (see Sarmet et al. 2024, for details).

### 2.3 Experimental Apparatus

**Figure 1:**
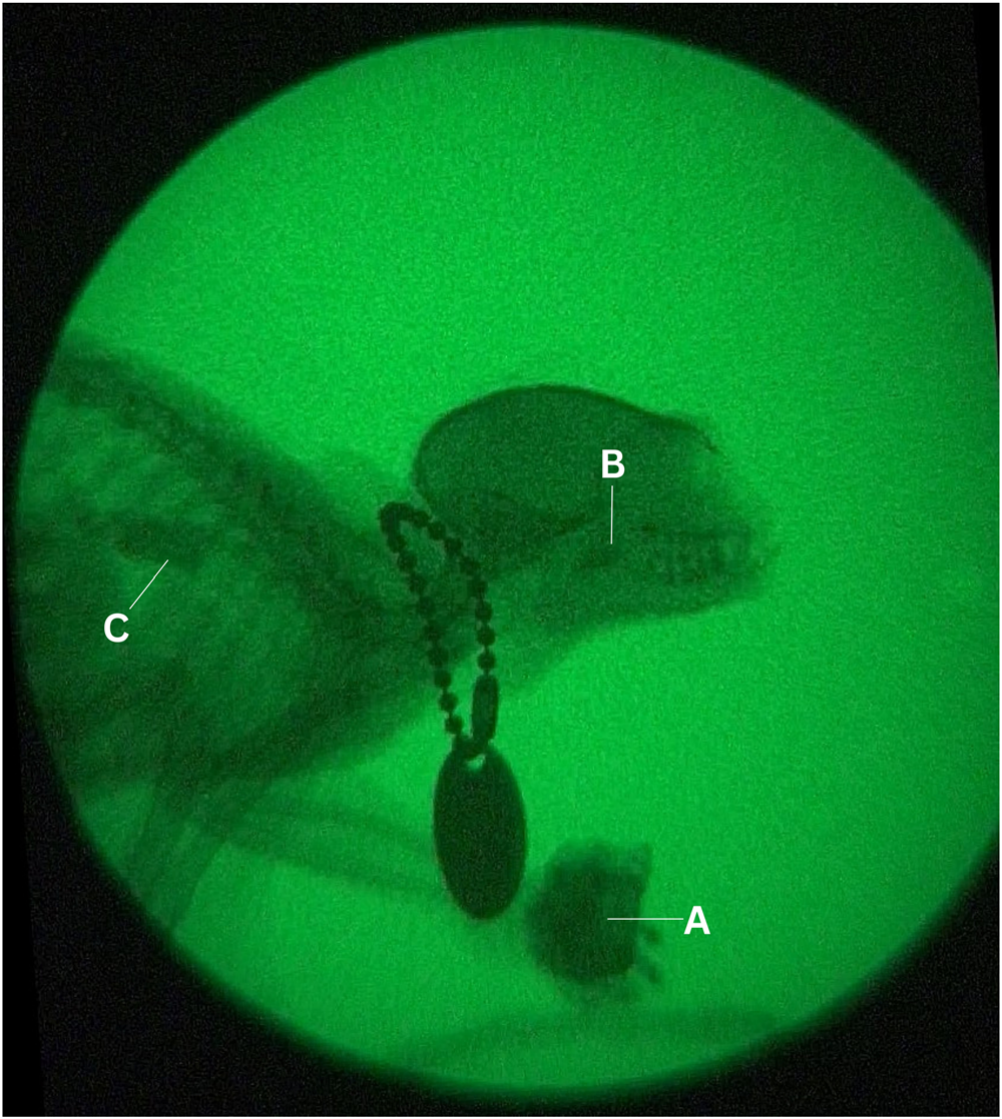
Example of a cineradiographic image of a 13‐year‐old female marmoset (M8). (A) Food portion; (B) food bolus in the oropharynx; (C) food bolus in the distal part of the esophagus. Image is cropped for clarity.

A comprehensive description of the use of cineradiography can be found in de Oliveira et al. (2022). In short, cineradiography is a dynamic radiological exploration technique developed by our research group in a reduced‐size apparatus adapted for small animals (Hasegawa et al. 2014; de Campos et al. 2015; de Campos et al. 2018; Kawamura et al. 2024). The cineradiographic apparatus (Micro X‐movie; NIC) transmitted an X‐ray beam vertically emitted to the marmoset by a microfocus X‐ray tube (Toshiba Electron Tubes and Devices Co. Ltd.) (Figure 1). To obtain stable X‐ray emission, we kept the power settings on the X‐ray tube constant at 70 kV and 0.3 mA. A beryllium fast‐response image‐intensifier (E5889BP‐P1K; Toshiba Electron Tubes and Devices Co. Ltd.) converted the X‐ray photons passing through the marmoset into visible light (de Campos et al. 2018; Kawamura et al. 2024). A digital video camera positioned underneath the image intensifier then captured this image (de Campos et al. 2018) and also recorded the audio of the experiments. The camera recorded video at 29.97 fps and 1920 × 1080 pixels.

### 2.4 Experimental Procedure

**Figure 2:**
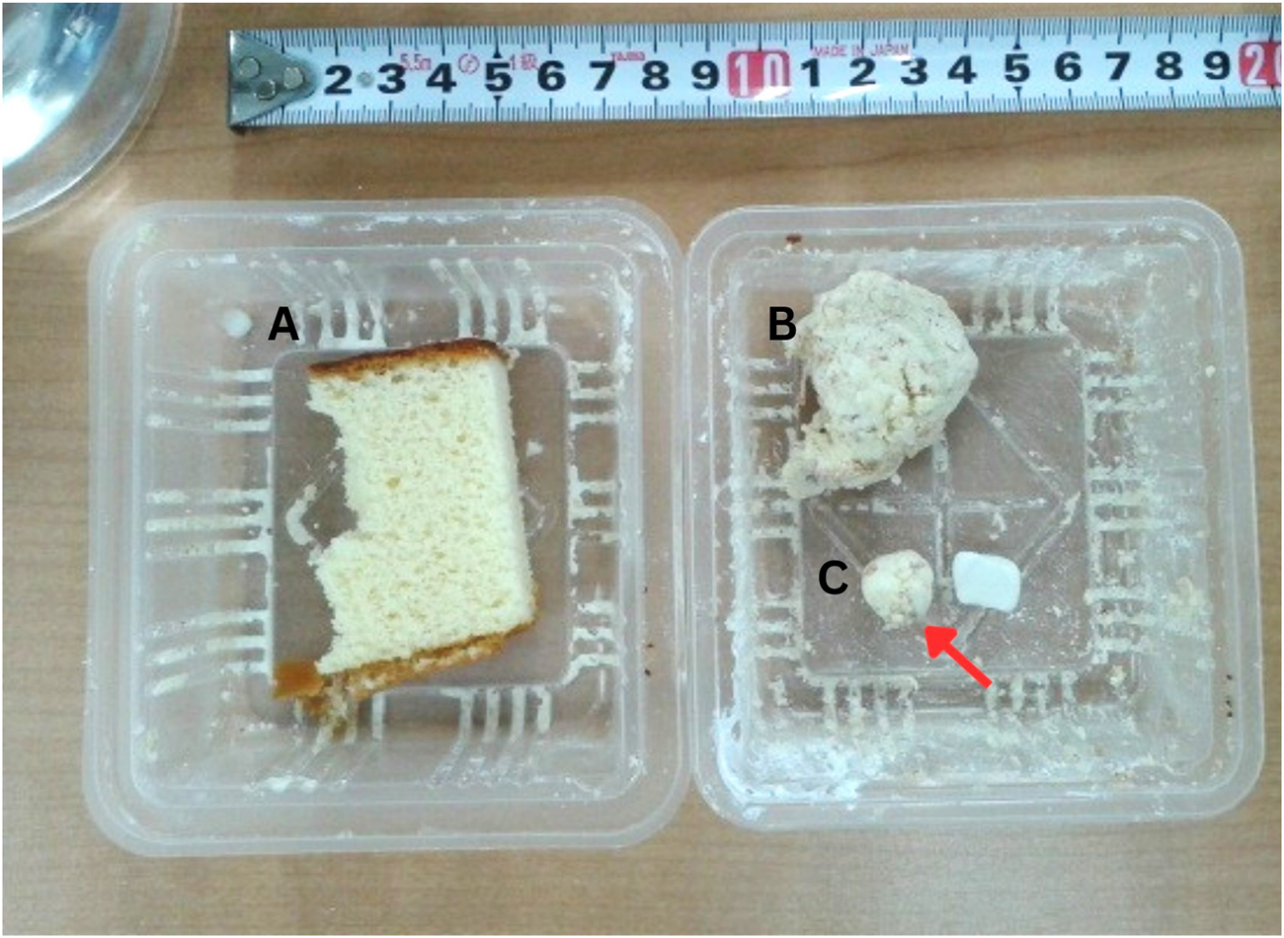
Example of the food offered to the animals. (A) Whole Castella cake in its unaltered form; (B) Smashed Castella cake mixed with barium sulfate prepared to be portioned; (C) A serving (portion) allocated for the marmosets' consumption (red arrow).

J. L. L. Z., P. S. C., K. K., and Y. K. conducted the recordings from January 2015 to April 2017 (infants/adults) and July–August 2017 (elderly). M. S. performed the image analyses (2022–2024). We individually transported marmosets from their home cages to the experiment room (around 7:00 am) and manually positioned them within the cineradiographic apparatus for 5‐min X‐ray sessions. We placed small barium‐mixed Castella cake balls, a preferred enrichment food at the facility, on the cage floor (IDDSI Level 6; Cichero et al. 2017; Figure 2). We stopped each session at 5 min, regardless of food consumption, after which we returned animals to their home cages. Despite the very low radiation dose emitted by cineradiography, a veterinarian examined all animals after the experiment for potential health issues related to X‐ray exposure.

### 2.5 Cineradiographic Recordings

**Figure 3:**
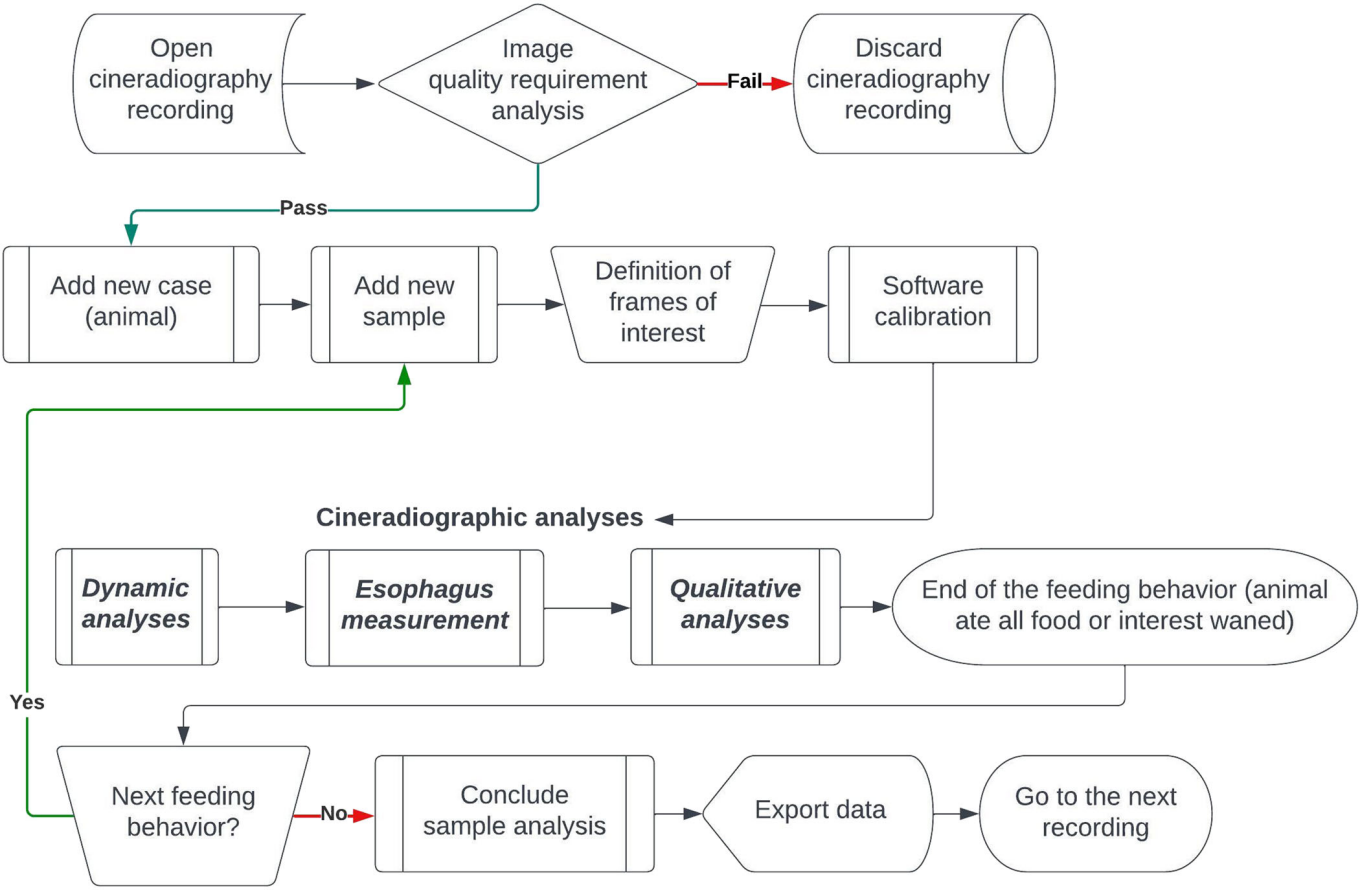
Flowchart of the cineradiographic analyses. Flowchart demonstrating how, from a single recording session for a single animal, multiple samples (feeding events) can be extracted for analysis.

Within each 5‐min recording, we collected multiple feeding sequences (chewing or swallowing; Figure 3) when animals showed sustained interest in the food. We recorded infants once between 1 and 4 months of age, with the exception of marmoset F1, which we also filmed at 1.5 and 2 years and included in both the infant and adult groups (Material S2). We filmed older animals once at a single age (Material S3). To ensure accurate intake measurement, we limited analyses to sequences providing a complete lateral view of the initial food portion, which was necessary for accurate determination of portion size. We analyzed only recordings meeting the criteria of observable feeding behavior, a lateral view, and a visible, measurable food portion, archiving all others. For each such analyzed sequence, we calculated net eating time, pausing the analysis when the animal's interest waned and resuming if feeding recommenced.

**Figure 4:**
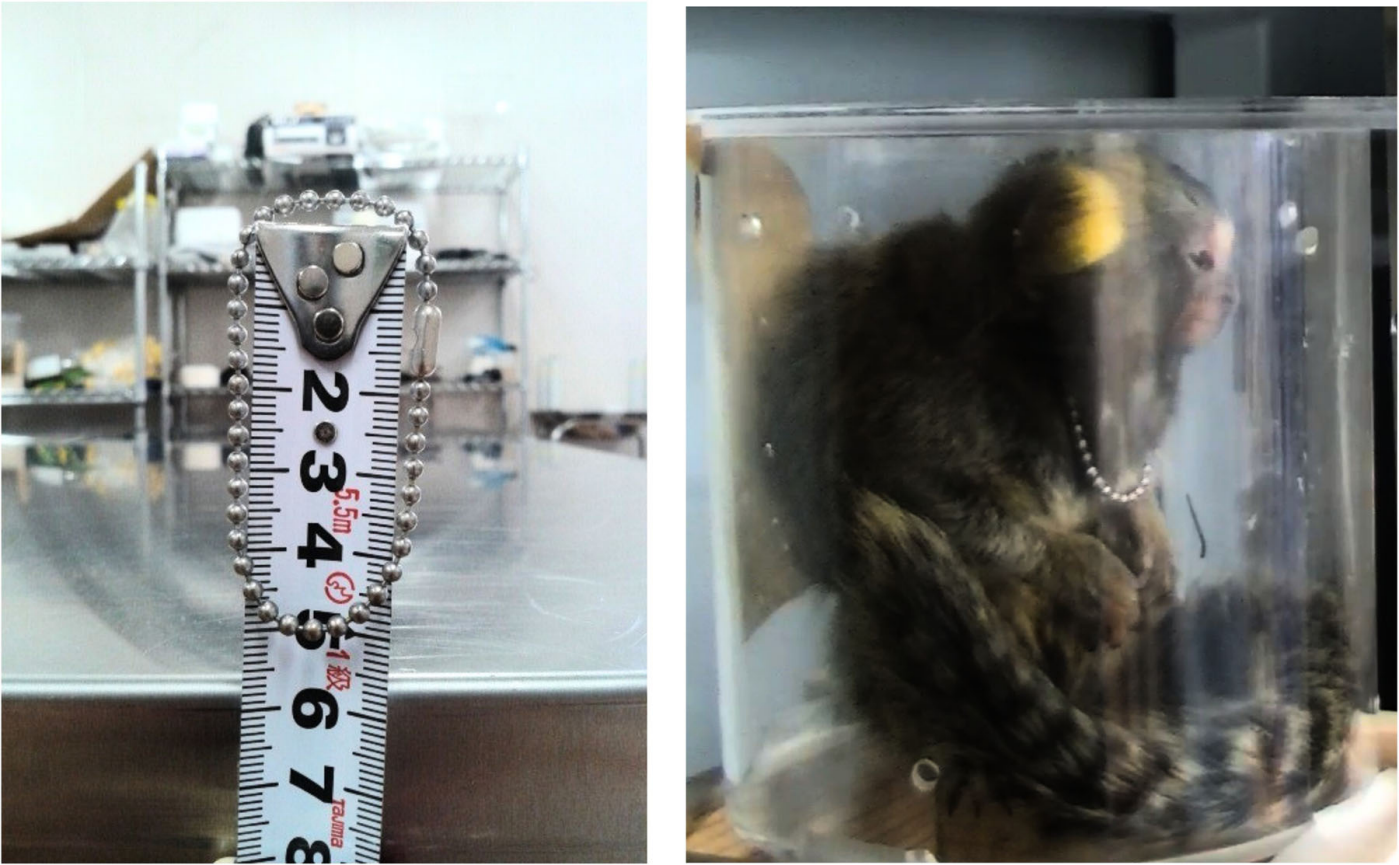
Calibration setup. This figure shows the calibration setup for the experiment. The left side depicts the stainless steel ball chain collar used for calibration purposes. The right side shows the test cage containing Marmoset F6, a 3‐month‐old female, wearing the collar.

We analyzed selected video recordings for feeding variables (Sections 2.6, 2.7, 2.8; Table 1) and qualitative behavioral characteristics (Section 2.9) using VLC Media Player 3.0.16 and the Time v3.2 extension (Mederi). We then exported frames of interest in JPEG to ImageJ (v. 1.53e) and calibrated them using a 2.5 mm stainless steel ball chain collar (Figure 4).

**Table 1:** Details of the definitions, measurements, and description of the experimental setup.

Variable and unit	Timing of measurement	Measurement definition	Description of the experimental setup
Esophageal caliber (mm)	At feeding behavior start	Maximum width of the esophagus when distended with food	Frame selected when the animal was in exact lateral view for accurate measurement
Portion size (mm^2^)	Right before the first masticatory cycle	The cross‐sectional area of the food portion measured just before the first chewing cycle	If the initial view is imperfect, we discard all feeding behavior until the measurable portion achieved
Masticatory cycles (n)	At feeding behavior start	Number of chews. A masticatory cycle was defined as (1) initial mandibular opening, (2) maximum mandibular opening, and (3) jaw closing	Counting performed at the end of each cycle
Duration of oral preparatory phase (s)	At feeding behavior start	Time from the first bite to the start of the first swallow	Calculated from the frame difference between two frames of interest
Bolus cross‐sectional area (mm^2^)	At the swallow	Bolus outline for area and size determination	Measured by three trained researchers to ensure consistent measurements
Number of swallows (n)	During feeding behavior	Swallow onset is identified when food bolus consolidated in valleculae before passing epiglottis, ending when epiglottis returns to its neutral position after the bolus enters the esophagus (Howe et al. 2023)	Total swallow count calculated for each feeding behavior
Esophageal transit time (s)	After the last swallow until the esophageal emptying	ETT onset was defined as the time from the final swallow event when the bolus entered the esophageal inlet to the frame, capturing the bolus' complete passage into the stomach (Garand et al. 2020)	For this analysis, the animal's position on the screen is irrelevant as long as the bolus transit through the esophagus is observable

The cross‐sectional area (mm²) of the visible food portion in the initial frame served as an estimate of food quantity consumed in each recording. Table 1 describes other measurements. Figure 3 shows the complete analysis process. We analyzed cineradiographic images for masticatory and swallowing function (Sections 2.6, 2.7, 2.8), performing temporal calculations based on identified initial and final frames and a 29.97 fps framerate, using the formula (final frame – initial frame)/29.97 to obtain durations in seconds.

### 2.6 Assessment of the Masticatory Function

We quantified masticatory cycles (initial opening, maximum gape, and jaw closure; de Oliveira et al. 2022) from video segments of constant feeding behavior using the Tap Counter 2.4 app (Android) at 50% speed. Based on these cycles and total chewing time, we calculated masticatory frequency (cycles/s), averaging the result per recording per animal. We then calculated the food portion area to masticatory cycle ratio (portion/mastications).

### 2.7 Assessment of the Swallowing Function

Using the same method as for masticatory cycles, we quantified swallows by identifying swallow onset at bolus consolidation in the valleculae before epiglottis passage and offset at epiglottis return after esophageal entry (Howe et al. 2023). For each sample, we counted the absolute number of these quantified swallows and averaged the results (Table 1). We then used these average swallow counts to calculate mastication and swallowing ratios for age‐group comparisons (Table 2).

**Table 2:** Derived ratio variables from feeding behavior analyses.

Variable	Calculation	Clinical interpretation
Portion rate (Portion/second)	Portion area (mm^2^)/net eating time	Higher values indicate a faster eating rate
Masticatory frequency (cycles/second)	Masticatory cycles/net eating time	Surrogate for masticatory speed; higher values indicate faster chewing
Portion/mastications	Ratio of portion/masticatory cycles	Higher values indicate reduced chewing effort
Portion/swallows	The ratio of portion/number of swallows	Higher values indicate fewer swallows per portion
Swallowing frequency (swallows/second)	Swallows/net eating time	Surrogate for swallowing speed; higher values indicate faster swallowing

To estimate bolus size during swallowing, we analyzed individual swallows from feeding videos and measured the bolus cross‐sectional area. A trained researcher identified frames where the bolus was clearly visible within the pharynx, just before entering the esophagus. These frames were saved in JPEG for further analysis. Three independent researchers then traced the bolus outlines using the freehand tool, and ImageJ was used to calculate the enclosed area as a measure of bolus area. The average value from the three independent tracings was used to define the bolus area for each swallow. The bolus area for each animal was defined based on their average during all the feeding sequence. This approach aligns with established protocols for bolus size estimation (Mayerl et al. 2023; Mayerl et al. 2020; Mayerl, Edmonds et al. 2021; Mayerl, Myrla et al. 2021; Ding et al. 2015; Edmonds et al. 2022). Measurement consistency was ensured using the intraclass correlation coefficient (ICC) (details below).

### 2.8 Esophageal Function Assessment

Following masticatory and swallowing analyses, we evaluated esophageal function using esophageal caliber (food‐filled esophageal diameter), esophageal transit time (ETT; time from final swallow to esophageal emptying), and esophageal clearance (MBSImP score: 0 = *clear*, 4 = *minimal/no clearance*; Martin‐Harris et al. 2008) to quantify esophageal residue.

### 2.9 Behavioral and Qualitative Analyses

In our qualitative assessment of marmoset feeding behavior, we evaluated four key parameters: feeding preference (manual or floor feeding), anterior food escape (spillage, indicating oral motor control), distraction (periods of nonchewing/swallowing), and vocalization (presence or absence).

### 2.10 Statistical Analyses

We conducted all statistical analyses using SPSS 29.0. Before main comparisons, we assessed normality with the Kolmogorov–Smirnov test. For age group comparisons, we used one‐way ANOVA with Bonferroni post hoc tests (95% CI) for normally distributed variables and Kruskal–Wallis tests with pairwise post hoc comparisons for nonnormally distributed variables. We also analyzed correlations using Pearson's correlation and linear regression and applied *χ* ^2^ tests for categorical variables. Regarding measurement reliability, we assessed bolus area measurement consistency using ICC (single‐rating, absolute agreement, two‐way mixed‐effects model; 95% CI), applying interpretation thresholds of > 0.90 (excellent), 0.75–0.90 (good), 0.5–0.75 (moderate), and < 0.5 (poor) (Koo and Li 2016). We additionally assessed the bolus size measurement scale's internal consistency using Cronbach's *α*. Statistical significance was set at *p* < 0.05.

## 3 Results

### 3.1 Animals

Table 3 summarizes the sample characteristics of the included marmosets (*n* = 26), including body weight, tooth status, and esophageal caliber.

**Table 3:** Sample characteristics (*n* = 26 marmosets).

Group	Number of animals	Sex (Male/Female)	Age in years (median, IQR)	Age in weeks (median, IQR)	Present teeth count (median, IQR)	Bleeding teeth count (median, IQR)	Body weight (g ± SD)^a^^,^ *	Post‐hoc comparisons	Esophageal caliber (mm) (median, IQR)^b^^,^ *	Post‐hoc comparisons
Infant	9	5♂ 4♀	0.2 (0.1)	12 (10)	28^d^	0	132.3 ± 47.5	infant < adult, old and very old*	4.1 (2.1)	infant < adult, old and very old*
Adult	2	0♂ 2♀	1.6 (1.3)	80 (64)	32^p^	0	306.0 ± 32.5		5.0 (0.9)	
Old	11	4♂ 7♀	12.2 (2.0)	586 (100)	11^p^ (14)	1 (3)	294.6 ± 59.4		5.3 (1.1)	
Very old	4	4♂ 0♀	16.1 (2.6)	775 (126)	9^p^ (6)	5 (6)	296.5 ± 37.0		5.6 (0.9)	

*Note:* The table reveals significant age‐related differences in body weight and esophageal caliber. Post‐hoc comparisons, adjusted using the Bonferroni correction, indicate that infants had significantly lower values for both measures compared with all other age groups. ♂ – male; ♀ – female.

Abbreviations: d, deciduous; p, permanent; IQR, interquartile range; SD, standard deviation.

^a^ ANOVA used for multiple group comparisons.

^b^ Kruskal–Wallis test used for multiple group comparisons.

* *p* < 0.001.

### 3.2 Recordings and Samples

This study analyzed 80 samples (feeding events) from 45 recordings of marmosets, resulting in the observation of 784 swallows. Table 4 summarizes the number of recordings, samples, observed swallows, and feeding durations for each age group. Twenty‐four recordings were excluded due to lack of feeding behavior during the recording (*n* = 18) or inadequate lateral visualization (*n* = 6). The number of recordings and samples for each individual, along with their mean feeding time, are provided in Material S2 (for the infant group) and Material S3 (for the adult, old, and very old groups).

**Table 4:** Sample characteristics by age group.

Age group	Number of animals (*n*)	Number of recordings (%)	Number of feeding samples (%)	Number of swallows observed (%)	Median feeding duration (s, IQR) (*p* = 0.99^a^)	Median net feeding duration (s, IQR) (*p* = 0.85^a^)
Infant	9	25 (55.5%)	42 (52.5%)	460 (58.6%)	47.00 (54)	32.00 (30)
Adult	2	5 (11.1%)	5 (6.2%)	61 (7.7%)	77.01 (132)	58.94 (86)
Old	11	11 (24.4%)	25 (31.2%)	215 (27.4%)	41.72 (20)	41.72 (23)
Very old	4	4 (8.8%)	9 (11.2%)	48 (6.1%)	53.50 (35)	41.00 (29)
**All**	26	45 (100%)	80 (100%)	784 (100%)	47.61 (44)	35.57 (27)

Abbreviations: IQR, interquartile range; s, seconds.

^a^ Kruskal–Wallis test used for multiple group comparisons.

### 3.3 Feeding Behavior and Physiology

Of 45 recordings, distraction occurred in 75.5% (*n* = 34), vocalization in 37.7% (*n* = 17), and anterior food escape in 24.4% (*n* = 11). Feeding was primarily manual (62.2%, *n* = 28) versus direct from the ground (35.5%, *n* = 16), with infants preferring ground feeding and adult, old, and very old marmosets preferring manual feeding (*χ*² = 14.35, *p* < 0.001). No significant age‐related differences were found for distraction (*χ*² = 0.17, *p* = 0.91), vocalization (*χ*² = 5.31, *p* = 0.15), or anterior food escape (*χ*² = 6.10, *p* = 0.11).

**Figure 5:**
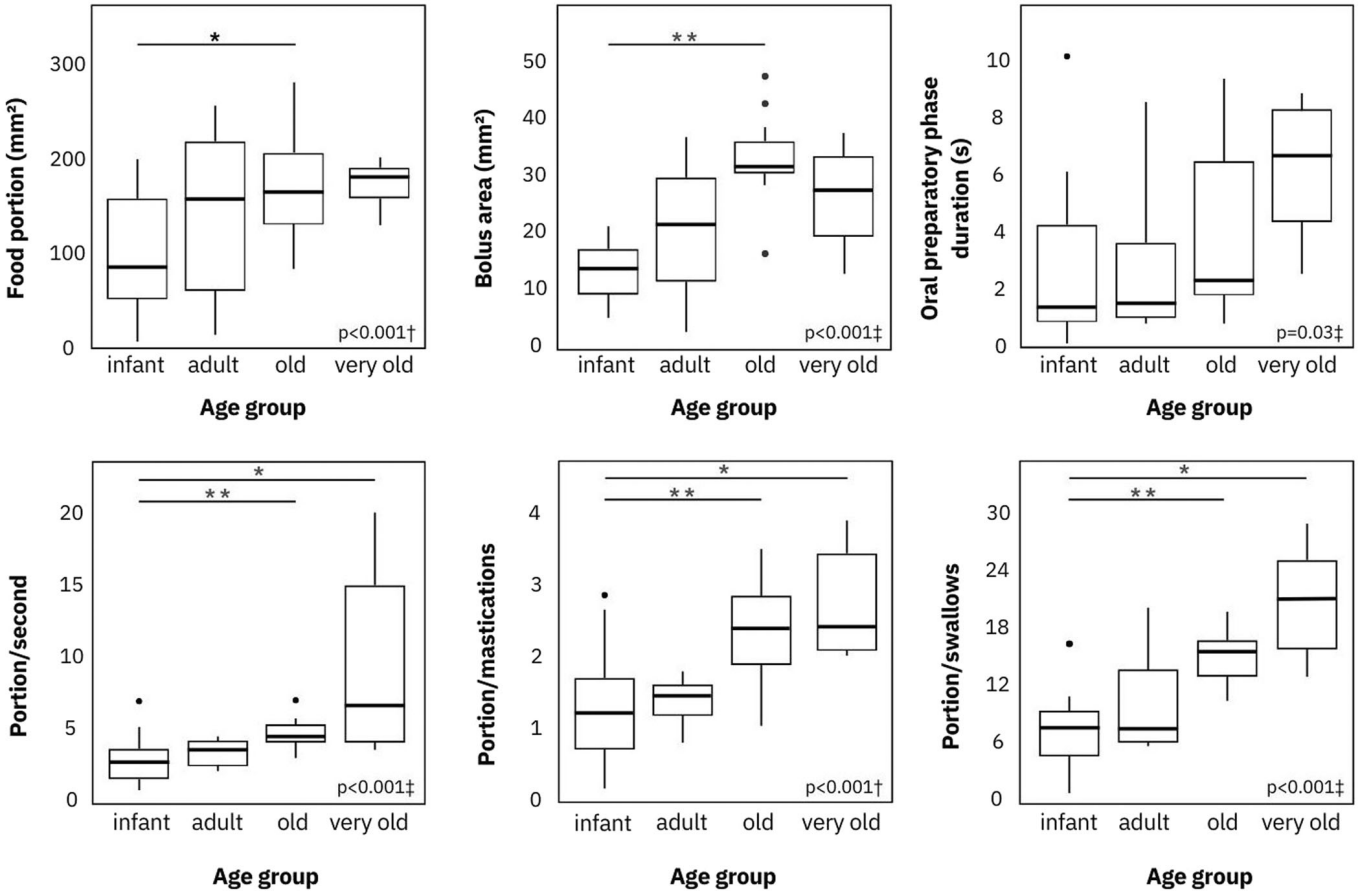
Box plots of key feeding behavior variables. Groups connected by lines were found to be significantly different based on post‐hoc comparisons with Bonferroni correction. * indicates *p* < 0.01 and ** indicates *p* < 0.001. ^†^ANOVA used for multiple group comparisons; ^‡^Kruskal–Wallis test used for multiple group comparisons.

**Figure 6:**
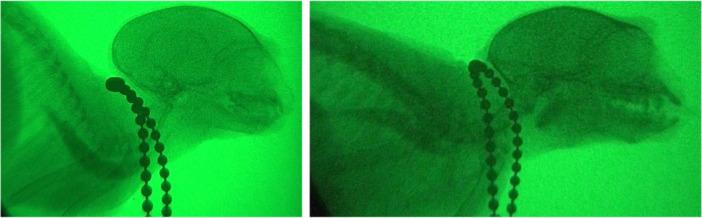
Esophageal retention. This figure showcases esophageal function in marmosets following food intake. Both the left image (2‐month‐old) and the right image (11‐year‐old) depict esophageal retention, as evidenced by the presence of food residue in the esophagus.

Old and very old animals exhibited longer oral preparatory phase durations, consumed larger food portions, and swallowed larger boluses with fewer mastications and swallows in shorter time intervals compared with younger animals. Excellent interrater reliability was achieved for bolus area measurements (ICC = 0.96, 95% CI: 0.95–0.96, *p* < 0.001; Cronbach's *α* = 0.98) and demonstrated high variability (Table 5, Figure 5). While ETT was not statistically significant due to high variability (*p* = 0.15, Kruskal–Wallis test, Table 5), a trend toward longer median ETTs was observed in old and very old animals. Median ETTs were as follows: infant 14.26 s (IQR = 10.31 s), adult 9.65 s (IQR = 3.58 s), old 14.44 s (IQR = 18.87 s), very old 20.39 s (IQR = 43.13 s). Esophageal retention (MBSImP score 1) was observed in all recordings (Figure 6). Correlations between feeding variables and age are presented in Material S4. Table 5 summarizes feeding dynamics, and Figure 5 depicts box plots of key feeding behavior variables, with significant group differences revealed by post‐hoc analyses. A comprehensive data set detailing the feeding variables, including range, measures of central tendency, and intergroup comparisons, can be found in Material S5.

**Table 5:** Summary of feeding behavior dynamics in marmosets (*n* = 26).

Variable and unit	Range	Mean/median	SD/IQR	*p* value
Food portion (cross‐sectional area, mm^2^)	6.54–268.80	127.46	69.47^†^	< 0.001
Duration of oral preparatory phase (s)	0.04–10.26	1.91	4.23^‡^	0.03
Masticatory cycles (*n*)	7–269	73.00	55.00^‡^	0.71
Masticatory frequency (cycles/second)	1.22–3.07	2.30	0.37^†^	0.99
Number of swallows (*n*)	1–42	13.00	7.00^‡^	0.78
Swallowing frequency (swallows/second)	0.08–0.67	0.37	0.15^†^	0.29
Bolus area (cross‐sectional area, mm²)	2.99–46.04	16.14	18.48^‡^	< 0.001
Esophageal caliber (mm)	2.06–6.12	4.67	1.66^‡^	< 0.001
Esophageal transit time (s)	5.13–114.89	14.31	12.05^‡^	0.15

*Note:* This table displays the frequencies of each variable observed in the recordings (*n* = 45) and the subsequent samples (*n* = 80) analyzed, encompassing data from the 26 animals included in the study.

Variables marked with “†” (normally distributed) are presented as mean ± standard deviation (SD). Variables marked with “‡” (nonnormally distributed) are presented as the median and interquartile range (IQR).

## 4 Discussion

Using cineradiographic recordings of multiple feeding events and swallows in marmosets across different age groups, this study investigated age‐related changes in feeding behavior. The findings revealed key differences in anatomy, mastication, and swallowing physiology, providing important baseline characteristics for captive marmosets.

Elderly marmosets (old and very old) exhibited significantly faster feeding rates, nearly quadrupling from infancy to very old age, consistent with trends observed in other primates (both platyrrhines and catarrhines). For example, faster feeding rates, increased intake, and decreased feeding time in older individuals have been reported in White‐faced capuchins (*Cebus capucinus*) (Mallott et al. 2017), Japanese macaques (*Macaca fuscata*) (Mori 1995; Jaman and Huffman 2011), and Rhesus macaques (*Macaca mulatta*) (Johnson et al. 1991). These findings, along with observations in other mammals (Chen et al. 2017; Schwermann et al. 2023), support the increased feeding rates we observed in elderly marmosets.

Elderly animals exhibited remarkably consistent feeding behavior, facilitating more detailed analyses. While all marmosets received identical food portions, variations in consumption necessitated a ratio‐based analysis for intergroup comparisons. Data collection was complicated by age‐related variations in feeding behavior. As expected, given their age (1–2 months) and developmental stage within the weaning period (solid food introduction around Day 30, within a ~75‐day weaning period; Oftedal et al. 2001; Power and Koutsos 2019; Brown et al. 2005), infant marmosets frequently interrupted feeding or showed disinterest, even shortly after recordings began. Conversely, elderly animals typically showed strong interest in the food, began eating promptly, and moved very little within the testing cage, simplifying measurements. In adults, however, high activity levels often obscured the food portion, requiring analysis to begin only when the food was fully visible and measurable.

Elderly marmosets exhibited faster feeding rates partly due to adaptations in their feeding behavior. The oral preparatory phase significantly increased with age, from 1.4 s in infants and adults to 2.3 s in the old and 6.4 s in very old individuals. This extended oral processing likely compensates for age‐related dental changes, as older animals consumed larger food portions and subsequently swallowed larger boluses, necessitating more extensive oral manipulation. This increased bolus size, however, has potential implications for swallowing safety. Despite similar body weights, elderly marmosets ingested larger boluses than adults, potentially increasing aspiration risk (Mayerl, Myrla et al. 2021; Chen et al. 2021; Setzen et al. 2003). However, because we did not use a liquid consistency—which is more readily aspirated in mammals and easier to observe—we cannot definitively confirm this possibility. Beyond aspiration risk, reduced mastication and larger boluses in elderly marmosets likely compromise digestion. Insufficient chewing reduces food surface area for enzymatic action, impairing digestion (Cassady et al. 2009). As suggested by human studies, this impaired digestion can decrease nutrient bioaccessibility and absorption (Cassady et al. 2009; Wang et al. 2024), particularly concerning given age‐related digestive decline (Bhutto and Morley 2008). Furthermore, poorly masticated, larger particles may delay gastric emptying, cause gastrointestinal discomfort, or exacerbate digestive issues (Yang et al. 2023), ultimately impacting their gut health and nutritional status in aging.

This increased bolus size may represent a compensatory mechanism to maintain feeding efficiency despite age‐related declines in masticatory ability, potentially reducing the number of swallows and overall feeding duration. This hypothesis is supported by the finding that feeding efficiency, measured as portion size relative to the number of mastications, increased with age (Figure 5). While masticatory frequency remained relatively stable across age groups (infants: 2.29; adults: 2.56; old: 2.19; very old: 2.40; *p* = 0.99) and showed no significant correlation with the number of present or bleeding teeth, the slightly higher frequency observed in very old marmosets might suggest a compensatory mechanism. This increased chewing activity in very old individuals could be an adaptation to overcome potential challenges associated with age‐related changes in oral health, such as tooth loss and periodontal disease. This finding supports the idea that elderly marmosets employ alternative strategies, such as the extended oral preparatory phase and larger bolus sizes, to maintain efficient food processing and minimize feeding time, potentially reducing reliance on extensive chewing.

Consistent with the increased masticatory efficiency, swallowing efficiency also increased with age. Very old marmosets required nearly three times fewer swallows than infants to consume the same amount of food, with adults and old marmosets exhibiting intermediate values. Although swallowing frequency did not differ significantly between age groups (*p* = 0.29), a trend toward decreasing frequency with age was observed (infants: 0.38 swallows/s; adults: 0.42; old: 0.34; very old: 0.28). This decrease in elderly marmosets also likely reflects the extended oral processing of larger food portions and reduced chewing efficiency, resulting in larger boluses. A more balanced distribution of individuals across age groups might reveal a statistically significant difference.

The observed feeding patterns in elderly marmosets suggest age‐related swallowing difficulties, or presbyphagia, consistent with those reported in aging humans. Presbyphagia refers to age‐related changes in swallowing that are part of normal aging and reflect neural and muscular degeneration (Ambiado‐Lillo 2024; Mancopes et al. 2021). To our knowledge, this is the first animal study to present data on age‐related changes in swallowing physiology in a nonhuman primate (NHP), making it difficult to determine if this phenomenon occurs in other NHP species.

Based on findings from human aging research, this difference in marmoset feeding physiology likely results from a combination of factors, including a potential age‐related decline in oropharyngeal sensorimotor function (Arce‐McShane et al. 2014; Nakamura et al. 2017). This decline may involve reduced somatosensory feedback from upper airway structures (Cole et al. 2023), possibly due to decreased taste bud density (Cole et al. 2023; Kano et al. 2007; Shimizu 1997) and blunted mechanosensory perception resulting from decreased pharyngeal and supraglottic innervation (Cole et al. 2023; Martin et al. 1994; Setzen et al. 2003). Additionally, non‐feeding‐related sensory declines, such as in visual and auditory systems, may contribute to altered sensory processing and the observed changes in feeding behavior (Shune and Moon 2016; Bailoni and Cerchiaro 2005). It is difficult to definitively confirm whether these specific mechanisms also occur in marmosets, as studies demonstrating the direct impact of aging on swallowing function have been conducted primarily in humans.

This study also expands knowledge of marmoset esophageal function by measuring esophageal caliber and motility during feeding. The median esophageal diameter (0.46 cm) was similar to that reported for Black‐tufted marmoset (*Callithrix penicillata)* (0.50 cm; Guimarães‐Lopes et al. 2020), validating cineradiography as a minimally invasive measurement technique. Given the limited data on esophageal function in *Callithrix*, the relatively slow esophageal emptying observed in all marmosets suggests a potential species‐specific trait. While median ETT did not vary significantly between age groups (overall median 14 s; infants: 14 s, adults: 9 s, old: 14 s, very old: 20 s), a trend toward increased ETT in very old marmosets was observed. Two individuals (one old, 114 s; one very old, 67 s) exhibited markedly prolonged ETTs. This variability, likely obscured by unbalanced group sizes, may indicate age‐related changes in esophageal function, warranting further investigation into potential age‐related esophageal remodeling, as described in other mammals (Zhao and Gregersen 2015; Bellows et al. 2016; Pathak et al. 2024; Abdelghani et al. 2023).

### 4.1 Implications

Our study revealed a developmental trajectory in marmoset chewing and swallowing behaviors, from infancy to old age, characterized by progressively refined handling of larger food portions and boluses. These evolving functions, which continued to change throughout the lifespan, likely reflect key developmental milestones. The observed differences between young and elderly marmosets likely result from a combination of learned compensatory strategies, selective pressures to minimize foraging effort (Barros et al. 2008; Teixeira et al. 2016; Ferrari and Beltrão‐Mendes 2011; Ferrari and Ferrari 1990), and potential age‐related sensorimotor deficits. Furthermore, the feeding strategies observed in the elderly marmosets in this sample could represent an evolutionary advantage contributing to their longevity. The feeding strategies observed in these exceptionally long‐lived senescent marmosets could represent either an evolutionary advantage contributing to their longevity or be a consequence of age‐related deficits, a distinction difficult to ascertain with the current sample lacking a comparative group of individuals who did not reach such advanced age. This novel, lifespan‐based analysis of marmoset food processing provides valuable insights into the feeding ecology and behavior of platyrrhine primates (Bezanson et al. 2024) and further supports their use as a model for studying human masticatory and swallowing dysfunctions.

### 4.2 Study Limitations

The results related to the infant group, containing animals with incomplete motor development, dependent on their mothers or even in the nursing phase, may have been compromised by separation from the mother at the time of the experiment, which could have increased their stress and influenced their motivation to feed. Also, the small sample size within the adult age group may have limited the statistical power of the analyses. Additionally, the absence of a thin liquid challenge in the swallowing assessment may have reduced the sensitivity for detecting subtle signs of aspiration and penetration. Future research should address these limitations by including larger sample sizes per age group and incorporating a thin liquid challenge into the swallowing protocol.

## 5 Conclusions

This study provides the first comprehensive analysis of masticatory and swallowing physiology across the marmoset lifespan, from infancy to very old age, significantly expanding our understanding of aging in this species. Our findings highlight distinct variations in feeding physiology across life stages. Notably, old and very old marmosets exhibited comparable masticatory and swallowing efficiency, suggesting the development of compensatory mechanisms in the oldest individuals to maintain functional feeding despite age‐related challenges. These feeding strategies may represent an evolutionary advantage contributing to their longevity. The consistent observation of esophageal retention across all age groups suggests this may be a normal physiological characteristic of the species. Overall, this study significantly contributes to our knowledge of marmoset anatomy, feeding behavior, and healthy aging, establishing baseline characteristics for the species and further supporting their use as a model for studying age‐related changes in human feeding and swallowing. Future research should investigate the underlying mechanisms driving these changes and explore their functional implications for overall health and well‐being in this well‐established aging model using NHP.

## Author Contributions

**Max Sarmet:** conceptualization, data curation, formal analysis, investigation, methodology, project administration, validation, visualization, writing – original draft (lead), writing – review and editing. **Sachiko Takehara:** funding acquisition, investigation, writing – review and editing. **Priscila Sales de Campos:** investigation, writing – review and editing. **Kensuke Kagiyama:** resources, investigation, writing – review and editing. **Yasuhiro Kumei:** funding acquisition, investigation, resources, writing – review and editing. **Christopher J. Mayerl:** supervision (supporting), writing – original draft (supporting), writing – review and editing. **Laura Davison Mangilli:** supervision (supporting), writing – original draft (supporting), writing – review and editing. **Jorge Luís Lopes Zeredo:** conceptualization, data curation, formal analysis, funding acquisition, investigation, methodology, project administration, resources, supervision (lead), writing – original draft (supporting), writing – review and editing (lead).

## Conflicts of Interest

The authors declare no conflicts of interest.

## Supporting information


**Supplementary Material 1:** Veterinary medical checkup schedule.

**Supplementary Material 2:** Number of samples collected and eating duration in the infant group.

**Supplementary Material 3:** Samples collected and eating duration ‐ adult, old and very‐old groups.

**Supplementary Material 4:** Table of correlations between the age, the number of present teeth, and the ratio variables.

**Supplementary Material 5:** Results of feeding dynamics among the age groups.

## Data Availability

Data sets, statistical code, and digital images used in analyses are available upon reasonable request.
