# Supplementary material for: Age‐Related Changes in Marmoset (Callithrix jacchus) Feeding Behavior and Physiology: Insights of Masticatory and Swallowing Functions
Source: Am J Primatol. 2025 Aug 26;87(8):e70070. doi: 10.1002/ajp.70070 (PMC12379082; doi:10.1002/ajp.70070)
Supplement: Supplementary file 1 — Supplementary Material 1: Veterinary medical checkup schedule. [file AJP-87-e70070-s002.docx]

**Supplementary material 1. Veterinary medical checkup schedule**

| **Test/assessment** | **Description** | **Examination interval** |
| --- | --- | --- |
| Behavioral health assessment | Signs like lethargy, unusual behaviors (such as excessive scratching), and significant weight fluctuations (either gain or loss) | Monthly |
| Gastrointestinal parasitology | Protozoan and helminth | Monthly |
| Body surface parasitology | Mites | Monthly |
| Microbial analysis of intestinal bacteria | Shigella Salmonella | Quarterly |
| Tuberculosis testing | Tubercle bacillus | Annually |
| Virus antibody test | Herpes simplex and measles | Annually |
| Multiple viruses test | Bunyaviruses, filoviruses, herpesvirus of turkey | Biannually |
| Other viruses | Simian Immunodeficiency, respiratory syncytial, Epstein‐Barr, Seneca Valley, Hepatitis B, and herpes simplex | Biannually |

Legend: This table describes the veterinary medical checkup assessments routinely performed on the marmoset colony at CLEA Japan.
