# Supplementary material for: Age‐Related Changes in Marmoset (Callithrix jacchus) Feeding Behavior and Physiology: Insights of Masticatory and Swallowing Functions
Source: Am J Primatol. 2025 Aug 26;87(8):e70070. doi: 10.1002/ajp.70070 (PMC12379082; doi:10.1002/ajp.70070)
Supplement: Supplementary file 2 — Supplementary Material 2: Number of samples collected and eating duration in the infant group. [file AJP-87-e70070-s001.docx]

**Supplementary material 2. Number of samples collected and eating duration in the infant group (n=9)**

| **Marmoset ID** | **Number of recordings** | **Samples per age** | | | |
| --- | --- | --- | --- | --- | --- |
|  |  | **1mo** | **2mo** | **3mo** | **4mo** |
| F1♀ | 4 | 2 | 1 | 1 | 1 |
| F3♂ | 3 | 4 | - | 1 | 2 |
| F4♂ | 4 | 2 | 1 | 1 | 2 |
| F5♂ | 3 | 2 | - | 1 | 2 |
| F6♀ | 3 | 3 | - | 2 | 1 |
| F7♀ | 3 | 4 | - | 2 | 1 |
| F8♂ | 1 | - | - | 1 | - |
| F9♀ | 3 | 2 | 1 | 1 | - |
| F10♀ | 1 | - | - | 1 | - |
| Total | 25 | 19 | 3 | 11 | 9 |
| Median net eating time, seconds (IQR) | | 31.00 (56) | 7.26 | 37.36 (27) | 28.95 (27) |

Legend: While all animals in the infant group were recorded between 1 to 4 months old, occasional instances occurred where they did not exhibit feeding behavior or interest in food. Consequently, these recordings were excluded. ♂ male, ♀female. IQR - Interquartile range
