# Supplementary material for: Age‐Related Changes in Marmoset (Callithrix jacchus) Feeding Behavior and Physiology: Insights of Masticatory and Swallowing Functions
Source: Am J Primatol. 2025 Aug 26;87(8):e70070. doi: 10.1002/ajp.70070 (PMC12379082; doi:10.1002/ajp.70070)
Supplement: Supplementary file 3 — Supplementary Material 3: Samples collected and eating duration ‐ adult, old and very‐old groups. [file AJP-87-e70070-s005.docx]

**Supplementary material 3. Samples collected and eating duration - juvenile (n=2), old (n=11) and very-old (n=4) groups**

| **ID** | **Number of recordings** |  |  | **Samples per age** | | | | | | |
| --- | --- | --- | --- | --- | --- | --- | --- | --- | --- | --- |
|  |  | **1yo** | **2yo** | **10yo** | **11yo** | **12yo** | **13yo** | **15yo** | **16yo** | **19yo** |
| F1♀ | 3 | 1 | 2 | - | - | - | - | - | - | - |
| F2♀ | 2 | 1 | 1 | - | - | - | - | - | - | - |
| M9♂ | 1 | - | - | 1 | - | - | - | - | - | - |
| S3♀ | 1 | - | - | - | 2 | - | - | - | - | - |
| M13♂ | 1 | - | - | - | 5 | - | - | - | - | - |
| M1♂ | 1 | - | - | - | 1 | - | - | - | - | - |
| M11♂ | 1 | - | - | - | 2 | - | - | - | - | - |
| M10♀ | 1 | - | - | - | - | 2 | - | - | - | - |
| S1♀ | 1 | - | - | - | - | - | 3 | - | - | - |
| S2♀ | 1 | - | - | - | - | - | 2 | - | - | - |
| M8♀ | 1 | - | - | - | - | - | 3 | - | - | - |
| M7♀ | 1 | - | - | - | - | - | 1 | - | - | - |
| M6♀ | 1 | - | - | - | - | - | 3 | - | - | - |
| M5♂ | 1 | - | - | - | - | - | - | 2 | - | - |
| M12♂ | 1 | - | - | - | - | - | - | - | 5 | - |
| M4♂ | 1 | - | - | - | - | - | - | - | 1 | - |
| M2♂ | 1 | - | - | - | - | - | - | - | - | 1 |
| Total | 20 | 1 | 3 | 1 | 10 | 2 | 12 | 2 | 6 | 1 |
| Net eating time (s) Median (IQR) | | 92.95 | 17.00 | 41.7 | 48.85 (14) | 26.0 | 33.67 (27) | 42.0 | 28.20 | 55.0 |

Legend: ID - identification; yo - years old; ♂ male, ♀female; s - seconds; SD - standard deviation.
