# Supplementary material for: Age‐Related Changes in Marmoset (Callithrix jacchus) Feeding Behavior and Physiology: Insights of Masticatory and Swallowing Functions
Source: Am J Primatol. 2025 Aug 26;87(8):e70070. doi: 10.1002/ajp.70070 (PMC12379082; doi:10.1002/ajp.70070)
Supplement: Supplementary file 4 — Supplementary Material 4: Table of correlations between the age, the number of present teeth, and the ratio variables. [file AJP-87-e70070-s004.docx]

**Supplementary material 4. Table of correlations between the age, the number of present teeth, and the ratio variables**

|  | Age | Present teeth | Masticatory frequency | Swallowing frequency | Portion/second | Portion/mastications | Portion/swallows | ETT |
| --- | --- | --- | --- | --- | --- | --- | --- | --- |
| Age | - | -0.90^**^ | -0.05 | -0.15 | 0.54^**^ | 0.62^**^ | 0.77^**^ | 0.24 |
| Present teeth | -0.90^**^ | - | 0.10 | 0.16 | -0.48^**^ | -0.59^**^ | -0.71^**^ | -0.15 |
| Masticatory frequency | -0.03 | 0.12 | - | 0.24 | 0.19 | -0.04 | -0.04 | -0.34^*^ |
| Swallowing frequency | -0.15 | 0.16 | 0.24 | - | 0.21 | 0.28 | -0.09 | -0.20 |
| Portion/second | 0.54^**^ | -0.48^**^ | 0.19 | 0.21 | - | 0.59^**^ | 0.47^**^ | 0.07 |
| Portion/mastications | 0.62^**^ | -0.59^**^ | -0.04 | 0.28 | 0.59^**^ | - | 0.80^**^ | 0.25 |
| Portion/swallows | 0.77^**^ | -0.71^**^ | -0.04 | -0.09 | 0.47^**^ | 0.80^**^ | - | 0.41^**^ |
| ETT | 0.24 | -0.15 | -0.34* | -0.20 | 0.07 | 0.25 | 0.41^**^ | - |

Legend: Age was measured in months old, present teeth in absolute numbers, masticatory frequency in cycles/second, swallowing frequency in swallows/second, and esophageal transit time in seconds. ETT Esophageal transit time; ^**^ p<0.01; ^*^ p<=0.05.
