# Supplementary material for: Age‐Related Changes in Marmoset (Callithrix jacchus) Feeding Behavior and Physiology: Insights of Masticatory and Swallowing Functions
Source: Am J Primatol. 2025 Aug 26;87(8):e70070. doi: 10.1002/ajp.70070 (PMC12379082; doi:10.1002/ajp.70070)
Supplement: Supplementary file 5 — Supplementary Material 5: Results of feeding dynamics among the age groups. [file AJP-87-e70070-s003.pdf]

Supplementary file 5. Results of feeding dynamics among the age groups (n=26)

	Infant (n=9, 42 samples)			Adult (n=2, 5 samples)			Old (n=11, 25 samples)			Very old (n=4, 9 samples)			
Variable	Range	Mean/median	SD/IQR	Range	Mean/median	SD/IQR	Range	Mean/median	SD/IQR	Range	Mean/median	SD/IQR	p
Food portion (mm^2)	6.54-205.00	96.34	60.91	11.00-252.00	142.92	101.82	88.40-268.80	172.72	50.53	142.00-204.70	181.99	27.47	†*
Oral preparatory phase duration (seconds)	0.04-10.26	1.41	3.57	1.08-8.65	1.48	5.83	1.17-9.72	2.37	5.60	2.35-8.80	6.40	5.56	‡*
Masticatory frequency (cycles/second)	1.22-2.87	2.33	0.59	2.27-3.07	2.46	0.63	1.60-2.77	2.13	0.47	2.07-2.67	2.43	0.54	‡0.46
Swallowing frequency (swallows/second)	0.08-0.67	0.40	0.27	0.23-0.67	0.40	0.18	0.20-0.47	0.37	0.16	0.13-0.57	0.30	0.38	‡0.82
Portion/second	0.39-7.42	2.19	2.11	1.84-4.51	3.54	2.33	2.92-7.54	4.48	1.32	3.72-20.75	6.86	13.67	‡**
Portion/mastications	0.21-3.16	1.23	1.04	0.89-1.81	1.55	0.70	1.17-3.45	2.41	0.69	2.20-3.84	2.76	0.74	†**

Portion/swallows	0.90-15.78	7.33	4.34	5.50-19.69	7.28	11.35	10.18-19.20	15.80	3.98	12.40-28.40	20.85	12.19	‡**
Esophageal transit time (seconds)	5.13-39.06	14.26	10.31	8.17-12.71	9.65	3.58	6.88-114.89	14.44	18.87	11.38-67.43	20.39	43.13	‡0.15

Legend:Animal F1, initially categorized within the infant group, was also recorded at 1 and 2 years old. To account for this developmental transition, we included its data from 1 to 4 months in the infant group analysis and its 1- and 2-year-old data in the adult group analysis. * - p<0.01; ** - p<0.001; † - ANOVA; ‡ - Kruskal-Wallis. Variables marked with † (normally distributed) are presented as mean ± standard deviation (SD). Variables marked with ‡ (non-normally distributed) are presented as the median and interquartile range (IQR).
